# Microsatellite markers for *Urochloa humidicola* (Poaceae) and their transferability to other *Urochloa* species

**DOI:** 10.1186/s13104-015-1044-9

**Published:** 2015-03-15

**Authors:** Jean CS Santos, Mariana A Barreto, Fernanda A Oliveira, Bianca BZ Vigna, Anete P Souza

**Affiliations:** Centro de Biologia Molecular e Engenharia Genética (CBMEG), Universidade Estadual de Campinas (UNICAMP), Cidade Universitária Zeferino Vaz, CP 6010, CEP 13083-875 Campinas, SP Brazil; EMBRAPA Southeast Livestock, Brazilian Agricultural Research Corporation, CP 339, São Carlos, SP CEP 13560-970 Brazil; Departamento de Biologia Vegetal, Instituto de Biologia, Universidade Estadual de Campinas (UNICAMP), Cidade Universitária Zeferino Vaz, CP 6109, CEP 13083-862 Campinas, SP Brazil

**Keywords:** Microsatellite, Genomic library, SSR transferability, Forage, Grass

## Abstract

**Background:**

*Urochloa humidicola* is a warm-season grass commonly used as forage in the tropics and is recognized for its tolerance to seasonal flooding. This grass is an important forage species for the Cerrado and Amazon regions of Brazil. *U. humidicola* is a polyploid species with variable ploidy (6X–9X) and facultative apomixis with high phenotypic plasticity. However, this apomixis and ploidy, as well as the limited knowledge of the genetic basis of the germplasm collection, have constrained genetic breeding activities, yet microsatellite markers may enable a better understanding of the species’ genetic composition. This study aimed to develop and characterize new polymorphic microsatellite molecular markers in *U. humidicola* and to evaluate their transferability to other *Urochloa* species.

**Findings:**

A set of microsatellite markers for *U. humidicola* was identified from two new enriched genomic DNA libraries: the first library was constructed from a single sexual genotype and the second from a pool of eight apomictic genotypes selected on the basis of previous results. Of the 114 loci developed, 72 primer pairs presented a good amplification product, and 64 were polymorphic among the 34 genotypes tested. The number of bands per simple sequence repeat (SSR) locus ranged from 1 to 29, with a mean of 9.6 bands per locus. The mean polymorphism information content (PIC) of all loci was 0.77, and the mean discrimination power (DP) was 0.87. STRUCTURE analysis revealed differences among *U. humidicola* accessions, hybrids, and other *Urochloa* accessions. The transferability of these microsatellites was evaluated in four species of the genus, *U. brizantha*, *U. decumbens, U. ruziziensis,* and *U. dictyoneura*, and the percentage of transferability ranged from 58.33% to 69.44% depending on the species.

**Conclusions:**

This work reports new polymorphic microsatellite markers for *U. humidicola* that can be used for breeding programs of this and other *Urochloa* species, including genetic linkage mapping, quantitative trait loci identification, and marker-assisted selection.

## Findings

### Background

*Urochloa humidicola* (Rendle) Morrone & Zuloaga (syn. *Brachiaria humidicola* (Rendle) Schweick.), commonly known as koronivia grass, is a perennial tropical grass native to eastern Africa that was introduced to Brazil in the 1950s [1,2]. *U. humidicola* is an apomictic polyploid species with variable levels of ploidy (6X–9X) [3-7].

In Brazil, the grasses of the genus *Urochloa* occupy 85% of the cultivated pasture areas [8]. *U. humidicola* is cultivated as forage in several tropical regions worldwide and is particularly recognized for its tolerance to poorly draining soils, seasonal flooding, and infertile acidic soils [9]. For this reason, this species has been largely exploited in the tropics as a forage option over other *Urochloa* grasses, mostly in the African savannas and similar environments, such as the Brazilian Cerrado [7].

The development and adoption of new *U. humidicola* cultivars with a broad genetic base are crucial for the diversification of forage pastures in the tropics, primarily because there are few cultivars of this species in Brazil (Tully, Llanero, and BRS Tupi). However, the development of new cultivars must be a dynamic process, providing cultivars with high nutritional value, increased biotic and abiotic resistance, and economic competitiveness.

Molecular markers are important tools to the progress of breeding programs, and their utilization would favor a more dynamic development of new cultivars of this species. However, there is a lack of information about the *U. humidicola* genome. Indeed, little or nothing is known about the number of genes, distribution of gene families, abundance and diversity of retro-elements, QTL localization of traits of economic importance, genome colinearity with model species, or abundance of repetitive sequences. Molecular markers are widely used in the fingerprinting of cultivars, the detection of genetic diversity in evaluating population structure in the mapping genes of interest, and in the selection of elite genotypes in breeding programs. SSR markers, in particular, are often used due to their codominant and multi-allelic characteristics [10]; moreover, they are highly site specific and transferable to related species [11].

Some microsatellite markers have already been developed for *U. humidicola* [12,13] and have been used for germplasm diversity studies [7,13], with all of them from the same microsatellite-enriched library constructed from genotype H016. Moreover, our research group identified four different gene pools among *U. humidicola* accessions; genotype H031 was found to be completely different from all other accessions, which was verified by a population structure analysis and by the fact that 18.5% of the tested markers did not amplify in this accession [7]. As a large number of markers are necessary for molecular breeding programs, our goal was to isolate and characterize new polymorphic microsatellite markers for *U. humidicola* genotype H031 (accession 12) to ensure that its genome was well represented by the new set of markers and also different accessions that belong to different gene pools and to test the transferability of these markers to four other *Urochloa* species (*U. brizantha*, *U. decumbens*, *U. ruziziensis*, and *U. dictyoneura*). The results were compared with previously reported data [12,13].

### Methods

The plant material for library construction and marker validation was obtained from young leaves from several *Urochloa* genotypes. For the first library (Lb-1) construction, a single sexual genotype (H031) was used. For the second library (Lb-2) construction, a pool of eight apomictic genotypes (H010, H013, H015, H034, H041, H043, H101, and H108) was used. For marker validation, 34 genotypes were selected, consisting of 20 *U. humidicola* germplasm accessions, six intra-specific hybrids, and eight *Urochloa* accessions, as represented by two different accessions from each of the following species: *U. brizantha*, *U. decumbens*, *U. ruziziensis*, and *U. dictyoneura*. These genotypes were selected based on the four gene pools found by a previous study [7], from which two genotypes were selected from each gene pool. All of the accessions used are from the *Urochloa* germplasm collection maintained at Embrapa Beef Cattle, Campo Grande, MS, Brazil. They have been personally identified by S. A. Renvoize, from the Royal Botanic Gardens, Kew, UK and their identity have been confirmed by C. B. do Valle when transferred to Brazil [9]. The annotation numbers, accession numbers (as recorded in Embrapa Beef Cattle (EBC) and Center for Tropical Agriculture (CIAT)), genotypes, and species identifications are shown in Table 1. Genomic DNA was extracted from freeze-dried leaf samples using the CTAB method [14]. The DNA samples were evaluated on a 1% agarose gel and quantified by comparison to known quantities of uncut λ phage DNA (Invitrogen, Carlsbad, CA, USA).

**Table 1:** **Genotypes of** ***U. humidicola*** **and four species of the genus** ***Urochloa*** **used for the characterization and transferability analyses of new microsatellite markers**

**AN**	**CIAT**	**BRA**	**EBC**	**Genotype**	**Species**
1	16181	4821	H004	germplasm accession	*U. humidicola*
2	16182	4839	H005	germplasm accession	*U. humidicola*
3	16867	4863	H006	germplasm accession	*U. humidicola*
4	16871	4901	H008	germplasm accession	*U. humidicola*
5	16880	4952	H010	germplasm accession	*U. humidicola*
6	16882	4979	H012	germplasm accession	*U. humidicola*
7	16886	5011	H013	germplasm accession	*U. humidicola*
8	26141	5088	H015	germplasm accession	*U. humidicola*
9	26149	5118	H016	germplasm accession	*U. humidicola*
10	16877	4928	H023	germplasm accession	*U. humidicola*
11	16894	5070	H030	germplasm accession	*U. humidicola*
12	26146	5100	H031	germplasm accession	*U. humidicola*
13	26413	6131	H035	germplasm accession	*U. humidicola*
14	26432	6203	H041	germplasm accession	*U. humidicola*
15	16884	4995	H044	germplasm accession	*U. humidicola*
16	NA	NA	H048	germplasm accession	*U. humidicola*
17	NA	1929	H107	germplasm accession	*U. humidicola*
18	6705	2208	H112	germplasm accession	*U. humidicola*
19	6133	1449	H125	germplasm accession	*U. humidicola*
20	6369	0370	H126	germplasm accession	*U. humidicola*
21	-	-	20	hybrid	*U. humidicola*
22	-	-	45	hybrid	*U. humidicola*
23	-	-	184	hybrid	*U. humidicola*
24	-	-	215	hybrid	*U. humidicola*
25	-	-	264	hybrid	*U. humidicola*
26	-	-	320	hybrid	*U. humidicola*
27	16162	-	B057	germplasm accession	*U. brizantha*
28	16467	-	B166	germplasm accession	*U. brizantha*
29	16499	004481	D009	germplasm accession	*U. decumbens*
30	26300	004707	D028	germplasm accession	*U. decumbens*
31	26163	005568	R102	germplasm accession	*U. ruziziensis*
32	26174	005614	R104	germplasm accession	*U. ruziziensis*
33	16186	007889	DT157	germplasm accession	*U. dictyoneura*
34	16188	007901	DT159	germplasm accession	*U. dictyoneura*

NA: not available, AN: annotation number, CIAT: Center for Tropical Agriculture, BRA: codes from EMBRAPA, EBC: codes from EMBRAPA Beef Cattle.

Genomic DNA was restriction digested with Afa I (Invitrogen), enriched in microsatellite fragments using (CT)8 and (GT)8 probes, and then used to construct a microsatellite-enriched library following the protocol of Billotte *et al*. [15]. The enriched microsatellite fragments were cloned into pGEM-T (Promega, Madison, WI), and the ligation products were used to transform *Escherichia coli* XL1-Blue competent cells. All 94 clones from both libraries were sequenced with an ABI 377 automated sequencer (Applied Biosystems, Foster City, CA) using the BigDye terminator cycle sequencing kit (Applied Biosystems, Foster City, CA).

The microsatellites were identified using MISA software [16]. Only mono-nucleotides with twelve or more repeats, di-nucleotides with six or more repeats, tri-nucleotides with four or more repeats, and tetra-, penta-, and hexa-nucleotides with three or more repeats were considered. Primer pairs were designed using the Primer Select 5.01 software (DNASTAR Inc.) and the Primer3Plus software [17]. Polymerase chain reactions (PCRs) were carried out as previously described [12]. The amplification products were resolved by electrophoresis through 3% agarose gels prior to vertical electrophoresis through 6% denaturing polyacrylamide gels. The gels were then silver stained [18], and the product sizes were determined by comparison to a 10-bp DNA ladder (Invitrogen, Carlsbad, CA).

Polyploid microsatellite genotyping is difficult due to the closeness of fragment sizes, stutter peaks observed and allele overlap due to multiple alleles of the same size. Few methods have been developed to overcome allele overlapping and estimate the allele frequencies, such as the estimation of alleles based on the electropherogram peak ratios [19] or the statistical estimation of allele frequencies [20]. However, for the present study work, we restricted the project to describe the new SSR markers, which were visually scored based on the presence (1) or absence (0) of a band in the polyacrilamide gels for each of the *Urochloa* genotypes. PIC (Polymorphic Information Content) [21] and DP (Discriminatory Power) [22] values were calculated to estimate polymorphisms at each locus.

The microsatellite scores for the 34 individuals were evaluated using a model-based method with Bayesian clustering approach in STRUCTURE software version 2.2 [23-25]. The admixture model was tested with 200,000 replicates for burn-in and 100,000 replicates for Markov Chain Monte Carlo (MCMC) processes through ten iterations (runs). The numbers of clusters (*K*) were tested from 2 to 20. The optimal number of clusters was estimated using the ΔK value, as previously described [26], and the final graphs were visualized using the STRUCTURE HARVESTER software [27]. The individuals were grouped into clusters according to the association coefficient (Q) proportion of each allelic pool in an individual.

A joint analysis (Lb-c) was performed with the data from the polymorphic loci derived from the new libraries Lb-1 and Lb-2. Data from a previous study [12] that used SSRs developed from accession 9 (H016) were used to compare the three libraries. The data were reanalyzed under the same parameters as those used for the new libraries, resulting in Lb-3. Another joint analysis (Lb-ct) was performed with data from the three libraries together (Lb-1, Lb-2, and Lb-3). The results obtained by STRUCTURE software were permuted by CLUMPP software [28], and the figures were generated using DISTRUCT software [29].

### Results

Microsatellite enrichment success for the *U. humidicola* DNA libraries was 79.0% for Lb-1 and 61.2% for Lb-2. From all of the sequenced clones, 183 microsatellites were identified. Di-nucleotide repeats were the most abundant class of microsatellites detected, representing 76.4% and 72.7% of the loci for Lb-1 and Lb-2, respectively, followed by mono-nucleotide and tetra-nucleotide repeats. Perfect microsatellites were the most abundant (Table 2).

**Table 2:** **Characterization of new microsatellite-enriched libraries from** ***U. humidicola***

**Library**		**Lb-1**	**Lb-2**
Total clones sequenced		86.0	80.0
Sequences containing microsatellites (%)		79.0	61.2
Total number of SSRs identified		106.0	77.0
*Type of repeat* (%)			
By nucleotide string	Mono-nucleotides	12.7	6.5
	Di-nucleotides	76.4	72.7
	Tri-nucleotides	1.9	5.2
	Tetra-nucleotides	5.6	11.6
	Penta-nucleotides	2.8	3.9
	Hexa-nucleotides	0.9	0.0
By form	Perfect	79.1	80.6
	Imperfect	9.3	1.6
	Perfect Compound	5.8	9.7
	Imperfect Compound	5.8	8.1

Of the 114 SSR primer pairs designed and tested, 72 were successfully amplified in *U. humidicola* genotypes, and 64 SSRs were polymorphic. A description of the number of alleles per locus and PIC and DP values for both the *U. humidicola* accessions and *Urochloa* accessions is presented in Table 3. The loci BhUNICAMP68 to BhUNICAMP108 are derived from Lb-1, and the loci BhUNICAMP109 to BhUNICAMP139 are derived from Lb-2. Based on the allelic frequencies estimated by STRUCTURE software, 36.43% of the alleles are rare (frequency < 0.05), 60.06% are intermediate alleles (0.05 < frequency < 0.30), and 3.50% are abundant alleles (frequency > 0.30).

**Table 3:** **Characterization of the 72 polymorphic SSR markers developed for** ***U. humidicola***

**SSR locus**	**GenBank accession number**	**Repeat motif**	**Ta (°C)** ^***a***^	**Primer sequences (5′-3′)**	***Urochloa*** species accessions*			***U. humidicola*** accessions**		
					**Size range (bp)**	**A** ^***b***^	**PIC** ^***c***^	**A** ^***b***^	**PIC** ^***c***^	**DP** ^***d***^
BhUNICAMP068	KM068303	(CACACC)_4_(CA)_17_	58.5	F_CCACAAACGTGAACACATACA R_AGGGACGGAAACACCCTTAG	226-261	10	0.87	10	0.87	0.95
BhUNICAMP069	KM068304	(TC)_25_	64.5	F_GAGGAACTCCTTTGGGTAGA R_TTCAGAGAGAGGATGGTATAGAG	285-300	2	0.36	2	0.36	0.58
BhUNICAMP070	KM068305	(GT)_9_	65	F_CCCCGGTCTCGACCTATC R_GAGGCTGCCCCCTTACTC	174-214	12	0.84	6	0.78	0.54
BhUNICAMP071	KM068306	(AC)_11_	65	F_CGCAACGAAGCTCCAATAG R_CGATCGCAAGCGTGTATCTA	160-228	11	0.86	11	0.86	0.94
BhUNICAMP072	KM068307	(GT)_7_	56.5	F_CCCCATGTAAACAACCGTAGA R_CCATGGTTGACCGCTAGAA	174-186	3	0.56	3	0.56	0.85
BhUNICAMP073	KM068308	(TG)_10_	60	F_TGAACATGTGAATGCCCACT R_ATTGCAGGATGCGGACTCTA	240-304	10	0.85	10	0.85	0.94
BhUNICAMP074	KM068309	(CT)_6_	58.5	F_ACGAACGATCCGACCAACTA R_TGCTTACGAGACGGCATAGA	231-255	7	0.81	7	0.81	0.92
BhUNICAMP075	KM068310	(TC)_22_	50	F_TGAATGCTTTTGTCCTGGTATC R_ACGTGCAGCAGCAACAGTA	148-236	28	0.95	24	0.95	0.98
BhUNICAMP076	KM068311	(AC)_18_	51.5	F_CCGATGGTCAAAGGTCAGTT R_GGTGGGCATATACCATGTTT	206-234	10	0.84	10	0.84	0.66
BhUNICAMP077	KM068312	(AC)_7_	65	F_CGGGAAGTCCTACTCCGTAA R_GGAGCTCAAGGTAGGGATTG	212-230	8	0.83	8	0.83	0.93
BhUNICAMP078	KM068313	(GT)_7_	58.5	F_ACCAGTGCACGTCTGAAAGA R_CGATCACTGCTGCGTCATA	216-218	2	0.35	2	0.35	0.52
BhUNICAMP079	KM068314	(AG)_12_G(GA)_17_	62.5	F_GGATTGAAAGTTGGAGCACA R_GCATGCTGTGAAGGAGGTTA	180-222	17	0.92	17	0.92	0.96
BhUNICAMP080	KM068315	(GA)_26_	50	F_CAAGCCTCTTCATGCAAGTAAC R_TGTCATACCCCCATGATTAAGA	176-230	22	0.93	21	0.93	0.93
BhUNICAMP081	KM068316	(AGC)_5_ACAAT(CA)_11_	55	F_CTGGCATGGGTCCCTTTAC R_TCTTCTTCCTCCAGCCACAT	160-179	5	0.75	5	0.75	0.95
BhUNICAMP082	KM068317	(CA)_23_	60	F_TTGCCGGGAACAGTTATACA R_GAAGCTCTATCAAACAGCCCT	157-192	9	0.82	9	0.82	0.92
BhUNICAMP083	KM068318	(AG)_22_	56.5	F_AAACATGCACCGTCATAACT R_GGGCTTGATTCATTTGTTA	152-190	6	0.68	4	0.68	0.77
BhUNICAMP084	KM068319	(TG)_15_	65	F_GGCGAAGACCATACCCTGTA R_TGCTGGTGGAAGAAGATGAA	159-182	9	0.80	9	0.80	0.96
BhUNICAMP085	KM068320	(GT)_9_	60	F_CGATTTATCGACGACCGAGT R_CCTTACTCGCAGGTCTGTCC	158-171	5	0.76	5	0.76	0.64
BhUNICAMP086	KM068321	(TC)_19_	65	F_AGTTGAATGGGCTGAACCAT R_TGCACTTCCAGGATCAGACA	238-326	10	0.82	10	0.82	0.93
BhUNICAMP087	KM068322	(GT)_10_	50	F_GGCCATTTCTAGCCAAACAA R_CCTTACTCGCAGGTCTGTCC	240	1	0.00	1	0.00	0.00
BhUNICAMP088	KM068323	(TG)_12_	65	F_AGAGGTTCCATGGACATTGC R_CTCATCAACAGACGCCTGAA	178	1	0.00	1	0.00	0.00
BhUNICAMP089	KM068324	(AC)_7_	65	F_CCGGATAGAAGGTCTGAACG R_AGTCGTCGAAGCGAGCTCTA	175	1	0.00	1	0.00	0.00
BhUNICAMP090	KM068325	(CA)_10_	65	F_CAGAGTAAGCTTCCGGGACA R_CGATTTATCGACGACCGAGT	200-300	12	0.85	11	0.85	0.91
BhUNICAMP091	KM068326	(AC)_8_	65	F_CTTGTGCCACTTCCACCTTT R_TCGTGTGGACACTTCCTCTG	120-150	9	0.83	9	0.83	0.95
BhUNICAMP092	KM068327	(TG)_6_	65	F_ATGCCTTGCTCCCACTAACA R_TAAATGCTCCAGCGACCTTC	135-168	11	0.85	11	0.85	0.91
BhUNICAMP093	KM068328	(AAG)_4_	65	F_GGAGCGCTAATTTCGTTCAG R_CCTCCGTTCTCGCTAATGAC	230	1	0.00	1	0.00	0.00
BhUNICAMP094	KM068329	(TG)_7_	65	F_TTGGAGCTTTCCCTAGCTCA R_GAACAAGAAGGGAGGAAGCA	272-290	4	0.31	4	0.31	0.39
BhUNICAMP095	KM068330	(TC)_16_(TG)_14_	65	F_GGGTTGGCCTACACACTGAT R_CGCACGACATTGATACCTTG	268-320	6	0.75	6	0.75	0.92
BhUNICAMP096	KM068331	(TC)_8_TT(TC)_40_	65	F_TGTTCTGCTCACTGGTTTGG R_TCAGCTCTCTACGGCTGGAT	157-255	11	0.87	11	0.87	0.95
BhUNICAMP097	KM068332	(GT)_6_	65	F_GCGAGCTACCGAGGTATTTG R_ACGTCAATGTCGAGCTTCCT	129-148	5	0.69	5	0.69	0.80
BhUNICAMP098	KM068333	(GT)_10_(G)_18_	65	F_GGACTGGTCGTCTTTCCATC R_GCTTTCTGCAAGCGGTAGAT	250-312	9	0.85	9	0.85	0.95
BhUNICAMP099	KM068334	(CA)_10_TG(GA)_10_	65	F_TTTGTGGCACCTGCAGAATA R_CGCTTCGTGCTGACAGATTA	124-174	16	0.91	16	0.91	0.99
BhUNICAMP100	KM068335	(TG)_12_	65	F_GCGCCATGGTTTCATCTATT R_GGTGGTTCCTCGTGTGAGAT	178-219	7	0.79	7	0.79	0.98
BhUNICAMP101	KM068336	(TG)_28_	65	F_GGTAAAGAAGGGCCGGACT R_GCATGGCATGTTCCTACTGA	128-184	14	0.89	12	0.89	0.97
BhUNICAMP102	KM068337	(GCGA)_4_	65	F_TGGTGGGCTCCACTATCTCT R_TCCGCCATCTCTCCTCTCT	224-260	12	0.89	12	0.89	0.94
BhUNICAMP103	KM068338	(CT)_22_	65	F_AGCTCTCCCGCCTCTCTCT R_CATCCACACCGTCTCTCTCA	100-156	14	0.91	14	0.91	0.96
BhUNICAMP104	KM068339	(TG)_26_	60	F_ACGACGACCTAATGGGTGAA R_ACCCAGCAACAAATCTCGTC	190-274	15	0.87	13	0.87	0.96
BhUNICAMP105	KM068340	(AC)_10_ATACACACACAC(AG)_53_	50	F_CTCCATCACGTGCTTGCTAA R_GTGTGATCGGCTGGAGATTT	100-176	30	0.93	29	0.93	0.98
BhUNICAMP106	KM068341	(TTTGT)_3_	50	F_GCTGTTCGGAGAGGAATCTG R_ATGAGAGGAGGGAAGGAAGG	135-155	8	0.79	7	0.79	0.91
BhUNICAMP107	KM068342	(GA)_18_	50	F_GGGTCAGTGTCGTCTCAGTTT R_CAGATTCCTCTCCGAACAGC	118-190	26	0.94	26	0.94	0.98
BhUNICAMP108	KM068343	(CT)_16_	65	F_TTGCCATTACTGGATCTGGA R_GCGCCACCCATAACTTAAA	112-160	14	0.85	13	0.85	0.94
BhUNICAMP109	KM068344	(GT)_9_	60	F_AGCGAGTCAAGCACAAGGAT R_GGGTCCAATCTCCCTCTCTC	186-226	9	0.82	9	0.82	0.93
BhUNICAMP110	KM068345	(TG)_8_	65	F_TCTGCATCCACTAGGCTCAG R_TCCTCCACCTTCTTTCCGTA	148-164	4	0.39	4	0.39	0.46
BhUNICAMP111	KM068346	(TG)_27_	65	F_AACTCCGACTATCTTCCAGTTGA R_AATGCATGGGTAGGATCTGC	250-330	15	0.89	15	0.89	0.96
BhUNICAMP112	KM068347	(AC)_26_	65	F_GACCAAACCCTCCGAAGTTA R_GGTTGCAACTACACGACCAG	246-300	10	0.81	10	0.81	0.94
BhUNICAMP113	KM068348	(CGTG)_3_	63	F_AACTTCGAGAGGTTCGTCCA R_ACCGGCAATCTATCCGTGT	144-179	3	0.45	3	0.45	0.51
BhUNICAMP114	KM068349	(CT)_21_	63	F_TATACAAGGCGCATCCACAA R_GCTCTTTCCTCACGCTGTTC	200-266	15	0.89	15	0.89	0.96
BhUNICAMP115	KM068350	(AC)_27_(AT)_7_	60	F_CTTCCTGCCAATAAGCGAAG R_CGAGCTTCCAGATTCTTTGG	240	1	0.00	1	0.00	0.00
BhUNICAMP116	KM068351	(TG)_8_	65	F_CTCCGCACCGCTTAAATTAG R_GTTGGAAATGGTGCTTCCAC	288-306	3	0.52	3	0.52	0.62
BhUNICAMP117	KM068352	(TGA)_7_	65	F_CCAACTGAACGGCCATACTT R_CCCACAAAGGAACCCTGAT	290-300	4	0.61	4	0.61	0.77
BhUNICAMP118	KM068353	(AG)_9_	50	F_CTGCATAACTTTCAGCCATCTC R_TTGGCACAACTGGAACGTAG	149	1	0.00	1	0.00	0.00
BhUNICAMP119	KM068354	(AAG)_7_	65	F_AAGGGCGTGATGTTCTGAAG R_AGGCCAAACGAATTTCTCAA	189-204	4	0.66	4	0.66	0.82
BhUNICAMP120	KM068355	(AT)_8_ACACACACACG(CA)_9_	65	F_TCCAGCAGTGTGTTCCTCAG R_ACCAGGAGTGCATAGCCAAG	190-200	6	0.71	6	0.71	0.75
BhUNICAMP121	KM068356	(TC)_12_	65	F_CGCTACTGCTGCACACAAAT R_CTGAGTGCGCCGTATGTTTA	170-195	6	0.71	6	0.71	0.92
BhUNICAMP122	KM068357	(GT)_15_	65	F_AGGAAGGCTCGCACTCACTA R_CCAAAGGCGGTGGTTAGATA	200-315	14	0.90	14	0.90	0.95
BhUNICAMP123	KM068358	(TTA)_4_	65	F_CCAAACTCTAGCTTTCACAGCA R_TTGGATCCACGTCAAACAAG	280	1	0.00	1	0.00	0.00
BhUNICAMP124	KM068359	(AG)_23_	65	F_TTGGAGTTGCTGGGCTATTT R_GAACCAAGCATAAGGCAACA	218-320	12	0.85	10	0.85	0.95
BhUNICAMP125	KM068360	(GT)_8_GAATGTGTGT(GA)_7_	65	F_TGTTATCAGTGCAGGTGTTGG R_GAGGCTGACGAAAGCTCAAC	258-280	7	0.81	7	0.81	0.93
BhUNICAMP126	KM068361	(AC)_10_	65	F_GGGAACCCAGGGTATCGTAT R_CTCTCCCAGCGTCTTTCCTT	210	1	0.00	1	0.00	0.00
BhUNICAMP127	KM068362	(GT)_6_	65	F_CCACCATTGCTTCCAGAGTAA R_ATTCGCCTCTCCTAGCACAA	272-320	7	0.69	7	0.69	0.91
BhUNICAMP128	KM068363	(GA)37	65	F_TGCCTGGAGACTGAGAAAGG R_CCTGCAGCAGACCTTCACAT	150-240	17	0.91	17	0.91	0.98
BhUNICAMP129	KM068364	(AC)_7_ATGAA(CATG)_3_(CA)_22_	63	F_TGTGTTTAGACCGCCAACAA R_TTATCGGCTCCCATTCACTC	207-310	11	0.84	10	0.84	0.95
BhUNICAMP130	KM068365	(AC)_7_	63	F_ACGCAGGAGAACTGCGTATC R_ATGGGATCCAACCGAACATA	236-300	12	0.79	11	0.79	0.87
BhUNICAMP131	KM068366	(AC)_7_(A)_16_	60	F_CATCAGATGCCTCAAACAGC R_GCAGGTGTGCAGCAAATAGA	184-238	14	0.87	14	0.87	0.93
BhUNICAMP132	KM068367	(TG)_7_(T)_29_	50	F_TCACTAGTGCGTCTGCTGCT R_GCACTCCATTGCAGACCTAAG	184-196	4	0.53	3	0.53	0.63
BhUNICAMP133	KM068368	(TG)_10_	50	F_CATGACTTATGTCCTTGGTGGA R_TCGACAGTGGAGCCACAA	114-162	19	0.89	16	0.89	0.97
BhUNICAMP134	KM068369	(CCGG)_3_	60	F_CAAACGGAGGAAGAGAGACG R_GGTGTCAATGCAGCCAAGTA	114-135	9	0.75	5	0.75	0.83
BhUNICAMP135	KM068370	(AG)_27_	65	F_CATGAGCCATCTCGTTGTTG R_TGCATTGACTTGACGTCTCC	176-260	14	0.90	9	0.90	0.91
BhUNICAMP136	KM068371	(AC)_9_(ACAA)_3_	50	F_TCCTGGTAAAGTTCCTCGTCA R_ACAACAATGCACGTCGAGAA	225-290	7	0.75	6	0.75	0.93
BhUNICAMP137	KM068372	(GA)_23_	65	F_TAGGTTTGGGTGGCACTAGG R_CTCCATGCTGCGTTGCTAT	258-320	11	0.85	9	0.85	0.91
BhUNICAMP138	KM068373	(T)_12_	60	F_TGCTCATGTGGGTCACATTT R_TGTGTGCCTGTGTGATGCTA	270-288	5	0.70	5	0.70	0.95
BhUNICAMP139	KM068374	(AAAAG)_3_	65	F_TCCTTTCTTTGAGCCGAGAG R_GCTGATGCTGACATCAAGGA	248-294	6	0.67	5	0.67	0.97
Total average						10.26	0.77	9.60	0.77	0.87
Lb-1 average						11.05	0.79	10.48	0.79	0.87
Lb-2 average						9.18	0.75	8.40	0.75	0.86

*Species evaluated: *Urochloa humidicola* (Rendle) Morrone & Zuloaga, *Urochloa brizantha* (Hochst. ex A. Rich.) R.D. Webster, *Urochloa decumbens* (Stapf) R.D. Webster, *Urochloa dictyoneura* (Figure & De Not.) Veldkamp, *Urochloa ruziziensis* (R. Germ. & C.M. Evrard) Crins.

**Hybrids included.

^*a*^Amplification temperature (°C).

^*b*^Maximum number of alleles observed.

^*c*^Polymorphism Information Content.

^*d*^Discrimination Power.

A survey of the potential transferability of the microsatellite markers from *U. humidicola* to other *Urochloa* species identified that 61.11% of the 72 markers resulted in amplified PCR products in at least one *U. brizantha* genotype, 58.33% were amplified in *U. decumbens*, 59.72% were amplified in *U. ruziziensis*, and 69.44% were amplified in *U. dictyoneura*. The number of successfully amplified genotypes per number of genotypes tested per species is shown in Table 4.

**Table 4:** **Cross-amplification of the 72 SSR markers among other** ***Urochloa*** **species**

**Transferability** ^***a,b***^				
**SSR locus**	***U. brizantha***	***U. decumben***	***U. ruziziensis***	***U. dictyoneura***
BhUNICAMP068	0/2	0/2	0/2	0/2
BhUNICAMP069	0/2	0/2	0/2	0/2
BhUNICAMP070	2/2	2/2	2/2	2/2
BhUNICAMP071	0/2	0/2	0/2	2/2
BhUNICAMP072	1/2	1/2	0/2	1/2
BhUNICAMP073	0/2	0/2	0/2	2/2
BhUNICAMP074	0/2	0/2	0/2	0/2
BhUNICAMP075	2/2	2/2	2/2	2/2
BhUNICAMP076	2/2	2/2	2/2	1/2
BhUNICAMP077	2/2	2/2	2/2	2/2
BhUNICAMP078	1/2	0/2	1/2	1/2
BhUNICAMP079	2/2	2/2	2/2	1/2
BhUNICAMP080	2/2	1/2	2/2	1/2
BhUNICAMP081	0/2	0/2	0/2	0/2
BhUNICAMP082	2/2	0/2	1/2	1/2
BhUNICAMP083	1/2	1/2	1/2	2/2
BhUNICAMP084	2/2	2/2	2/2	2/2
BhUNICAMP085	2/2	2/2	2/2	2/2
BhUNICAMP086	2/2	2/2	2/2	2/2
BhUNICAMP087	2/2	2/2	2/2	2/2
BhUNICAMP088	2/2	2/2	2/2	2/2
BhUNICAMP089	0/2	0/2	0/2	0/2
BhUNICAMP090	2/2	2/2	2/2	2/2
BhUNICAMP091	0/2	0/2	0/2	2/2
BhUNICAMP092	0/2	0/2	0/2	2/2
BhUNICAMP093	0/2	0/2	0/2	0/2
BhUNICAMP094	2/2	2/2	1/2	2/2
BhUNICAMP095	0/2	0/2	0/2	0/2
BhUNICAMP096	2/2	2/2	1/2	2/2
BhUNICAMP097	2/2	2/2	2/2	2/2
BhUNICAMP098	0/2	0/2	0/2	0/2
BhUNICAMP099	0/2	0/2	0/2	0/2
BhUNICAMP100	0/2	0/2	0/2	0/2
BhUNICAMP101	2/2	2/2	2/2	2/2
BhUNICAMP102	0/2	0/2	0/2	0/2
BhUNICAMP103	2/2	1/2	2/2	2/2
BhUNICAMP104	2/2	2/2	2/2	2/2
BhUNICAMP105	2/2	2/2	2/2	2/2
BhUNICAMP106	2/2	2/2	2/2	2/2
BhUNICAMP107	2/2	2/2	2/2	1/2
BhUNICAMP108	2/2	2/2	2/2	1/2
BhUNICAMP109	2/2	2/2	2/2	2/2
BhUNICAMP110	2/2	2/2	2/2	2/2
BhUNICAMP111	2/2	2/2	2/2	2/2
BhUNICAMP112	2/2	2/2	2/2	2/2
BhUNICAMP113	0/2	0/2	0/2	0/2
BhUNICAMP114	0/2	0/2	0/2	0/2
BhUNICAMP115	0/2	0/2	0/2	0/2
BhUNICAMP116	2/2	2/2	2/2	2/2
BhUNICAMP117	0/2	0/2	0/2	2/2
BhUNICAMP118	0/2	0/2	0/2	0/2
BhUNICAMP119	0/2	0/2	0/2	0/2
BhUNICAMP120	2/2	2/2	2/2	2/2
BhUNICAMP121	2/2	2/2	2/2	2/2
BhUNICAMP122	0/2	0/2	0/2	0/2
BhUNICAMP123	0/2	0/2	0/2	2/2
BhUNICAMP124	2/2	2/2	2/2	0/2
BhUNICAMP125	0/2	0/2	0/2	0/2
BhUNICAMP126	2/2	2/2	2/2	2/2
BhUNICAMP127	0/2	0/2	0/2	0/2
BhUNICAMP128	0/2	0/2	0/2	0/2
BhUNICAMP129	2/2	2/2	2/2	0/2
BhUNICAMP130	2/2	2/2	2/2	2/2
BhUNICAMP131	2/2	2/2	2/2	2/2
BhUNICAMP132	0/2	0/2	0/2	2/2
BhUNICAMP133	2/2	2/2	2/2	2/2
BhUNICAMP134	2/2	2/2	2/2	2/2
BhUNICAMP135	2/2	2/2	2/2	2/2
BhUNICAMP136	2/2	2/2	1/2	2/2
BhUNICAMP137	2/2	2/2	2/2	2/2
BhUNICAMP138	0/2	0/2	0/2	2/2
BhUNICAMP139	2/2	2/2	2/2	2/2
Total	44	42	43	50
Amplification %	61,11	58,33	59,72	69,44

^*a*^Number of successfully amplified genotypes/Number of tested genotypes.

^*b*^Nomenclatural classification: *Urochloa humidicola* (Rendle) Morrone & Zuloaga, *Urochloa brizantha* (Hochst. ex A. Rich.) R.D. Webster, *Urochloa decumbens* (Stapf) R.D. Webster, *Urochloa dictyoneura* (Figure & De Not.) Veldkamp, *Urochloa ruziziensis* (R. Germ. & C.M. Evrard) Crins.

**Figure 1:**
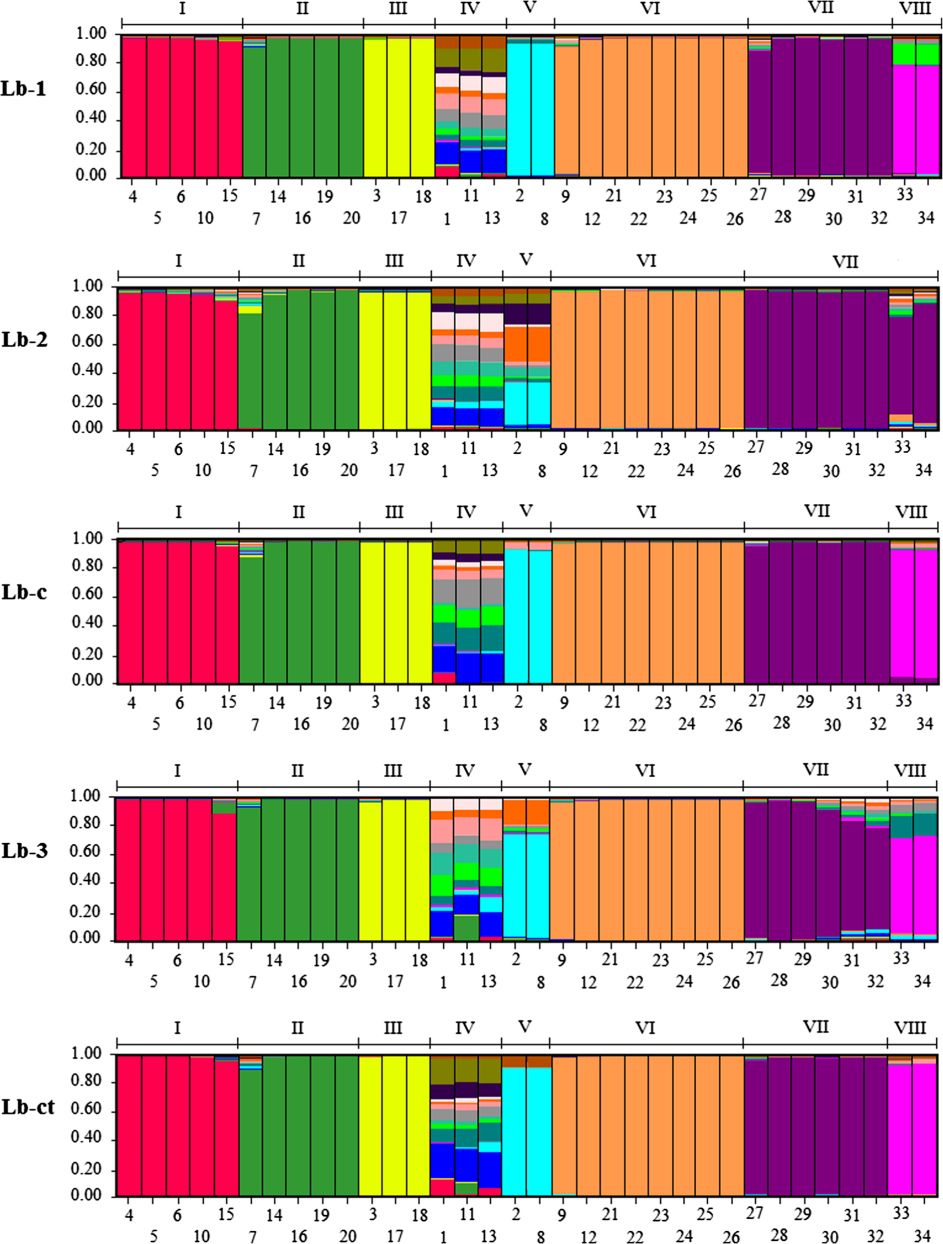
**Analysis performed with STRUCTURE software.** Lb-1: Library constructed from a sexual accession (H031), Lb-2: Library constructed from a pool of eight apomictic accessions, Lb-3: Library constructed from an apomictic accession (H016) [12], Lb-c: Joint analysis of Lb-1 and Lb-2, Lb-ct: Joint analysis of Lb-1, Lb-2, and Lb-3. Each of the 34 genotypes is represented by a single column divided into colored segments with lengths proportional to each of the allelic pools inferred by K through Evanno method [24]. Each K is represented by a different color and Lb-1 presented K = 18, Lb-2 K = 17, Lb-c K = 17, Lb-3 K = 15, and Lb-ct K = 18. The individuals were grouped into clusters according to the Q proportion of each allelic pool in an individual. Eight clusters were identified for Lb-1, Lb-c, Lb-3, and Lb-ct (I to VIII) and seven clusters for Lb-2 (I to VII). The left scale indicates the association coefficient (Q) for the assignment of genotypes into groups. The genotypes are named according to the annotated numbers listed in Table 1.

The population structure analysis based on SSR allelic data showed differentiation among the *U. humidicola* accessions, hybrids, and other *Urochloa* species. The STRUCTURE analysis for Lb-1 and Lb-2 and the joint analysis of data from both libraries (Lb-c) showed K = 18, K = 17, and K = 17 allelic pools, respectively, with each one represented by a different color in Figure 1. Clusters I to V were composed of *U. humidicola* accessions. Cluster VI was composed of two *U. humidicola* accessions (accessions 9 and 12) and six hybrids derived from a controlled cross between these two accessions. The other *Urochloa* species were grouped into Clusters VII and VIII for Lb-1 and Lb-c and in Cluster VII for Lb-2.

The STRUCTURE analysis for Lb-3 and Lb-ct showed K = 15 and K = 18 allelic pools, respectively (Figure 1), classified in the same clusters as for Lb-1 and Lb-c.

### Discussion

In the present study, we described 72 new SSRs for *U. humidicola*, 64 of which are polymorphic. Along with the 67 previous developed SSRs [12,13], these markers contribute to the genetic breeding of the species and other species of the genus *Urochloa* in efforts to obtain new cultivars and better understanding of the species genetic, through genetic mapping, marker-assisted selection, genome sequencing and synteny.

The increased occurrence of di-nucleotide motifs for Lb-1 and Lb-2 is in accordance with the enrichment of both libraries with (CT)_8_ and (GT)_8_ probes. In addition, Morgante *et al*. [30] reported a higher occurrence of microsatellites with di-nucleotide motifs in plants, which may have been a contributing factor in our observation.

Among the microsatellites analyzed, 88% had a polymorphism among the evaluated genotypes. The most informative loci in this panel of SSRs were those with the highest PIC and DP values (BhUNICAMP075 and BhUNICAMP107). Locus BhUNICAMP094 showed the lowest values for PIC and DP, at 0.3165 and 0.3969, respectively, even though it was amplified in all the *Urochloa* species evaluated. This also occurred with the BhUNICAMP030 locus [12]. Both loci may be useful markers for studies in *Urochloa* because it may be the result of a conserved region among the species studied herein. Monomorphic loci may be useful in other studies.

The transferability rates of the loci from *U. humidicola* to four other species were very similar. Although these results were not highly variable, *U. dictyoneura* presented the highest transferability, corroborating the genetic closeness between *U. dictyoneura* and *U. humidicola*, as has been previously described [2,31] and the results obtained in another study [13].

For the population structure analysis, different numbers of allelic pools [K] were observed for all analyses. However, the individual composition presented in each cluster was maintained into Lb-1, Lb-c, Lb-3, and Lb-ct analyses, but for Lb-2 analysis, the Clusters VII and VIII were grouped into Cluster VII.

The genotypes of the species *U. brizantha*, *U. decumbens*, and *U. ruziziensis* were grouped into the same cluster in all the analyses. However, the *U. dictyoneura* genotypes were grouped separately from the other species for all the analyses, except for Lb-2, with the four species grouping into Cluster VII.

In all analyses, Cluster VI included accessions 9 and 12, and six hybrids derived from crosses between these two accessions grouped together. However, in a previous study, the progenitors did not group together with the hybrids [13], as only runs from K = 1 to K = 10 were performed. These hybrids are part of an F_1_ population that is being mapped with the SSRs described in this study and previously published [12,13].

Although some discrepancies were found among the three libraries (Lb-1, Lb-2, and Lb-3), the set of loci belonging to each was able to satisfactorily differentiate the accessions. Comparing the three libraries developed, Lb-1 presented the highest number of allelic pools, which may be correlated to the usage of the accession H031, a highly diverse genotype, as described by [7]. The genotype used for the enriched library construction directly influences the results. The joint analysis of the three libraries (Lb-ct) would be the most recommended way to differentiate among accessions, because it uses loci derived from many different genotypes, conferring a greater reliability of the observed results.

These markers are immediately useful for *U. humidicola* breeding programs, aiding in areas such as the construction of linkage and QTL maps, gene flow and mating system evaluation, and marker-assisted selection.

## Availability of supporting data

The datasets supporting the results of this article are included in the article.

## Abbreviations

A: Maximum number of alleles observed
AN: Annotation number
BRA: Codes of the accessions from EMBRAPA
CAPES: Coordination of Improvement of Higher Education Personnel
CIAT: Center for Tropical Agriculture
CTAB: Cetyltrimethyl ammonium bromide
DNA: Deoxyribonucleic acid
DP: Discrimination power
EBC: Embrapa beef cattle
EMBRAPA: Brazilian Agricultural Research Corporation
FAPESP: Foundation for Research Support of the State of Sao Paulo
K: Number of clusters
Lb-1: Library construction from a sexual accession (H031)
Lb-2: Library construction from a pool of eight apomictic accessions
Lb-3: Library construction from an apomictic accession (H016) [12]
Lb-c: Joint analysis of Lb-1 and Lb-2
Lb-ct: Joint analysis of Lb-1, Lb-2, and Lb-3
MCMC: Markov Chain Monte Carlo
NA: Not available
bp: Base pairs
PCR: Polymerase chain reaction
PIC: Polymorphism information content
Q: Association coefficient from STRUCTURE analysis
QTL: Quantitative trait locus
SSR: Simple sequence repeat
Ta (°C): Annealing temperature

## References

[1] Sendulsky T (1978). *Brachiaria*: taxonomy of cultivated and native species in Brazil. Hoehnea.

[2] Renvoize SA, Clayton WD, Kabuye CHS. Morphology, Taxonomy and Natural Distribution of *Brachiaria* (Trin.) Griseb. In: Miles JW, Maass BL, Valle CB, editors. *Brachiaria*: Biology, agronomy, and improvement. Embrapa/CIAT; 1996. p.1-15.

[3] Adamowski EV, Boldrini KR, Pagliarini MS, Valle CB (2007). Abnormal cytokinesis in microsporogenesis of *Brachiaria humidicola* (Poaceae: Paniceae). Genet Mol Res.

[4] Boldrini KR, Pagliarini MS, do Valle CB (2009). Meiotic behavior of a nonaploid accession endorses x = 6 for *Brachiaria humidicola* (Poaceae). Genet Mol Res.

[5] Boldrini KR, Micheletti PL, Gallo PH, Mendes-Bonato AB, Pagliarini MS, do Valle CB (2009). Origin of a polyploid accession of *Brachiaria humidicola* (Poaceae: Panicoideae: Paniceae). Genet Mol Res.

[6] Boldrini KR, Pagliarini MS, do Valle CB (2010). Evidence of natural hybridization in *Brachiaria humidicola* (Rendle) Schweick. (Poaceae: Panicoideae: Paniceae). J Genet.

[7] Jungmann L, Vigna BBZ, Boldrini KR, Sousa ACB, do Valle CB, Resende RMS (2010). Genetic diversity and population structure analysis of the tropical pasture grass *Brachiaria humidicola* based on microsatellites, cytogenetics, morphological traits, and geographical origin. Genome.

[8] Moreira LM, Martuscello JA, Fonseca DM, Mistura C, Morais RV, Júnior JIR. Perfilhamento, acúmulo de forragem e composição bromatológica do capim-braquiária adubado com nitrogênio. In: Revista Brasileira de Zootecnia.2009. p. 1675–1684. http://www.scielo.br/pdf/rbz/v38n9/06.pdf. Accessed: 02 April 2014.

[9] Keller-Grein G, Maass BL, Hanson J. Natural variation in *Brachiaria* and existing germplasm collections. In: Miles JW, Maass BL, Valle CB, editors. *Brachiaria*: biology, agronomy and improvement. Embrapa/CIAT; 1996. p.16-42.

[10] Rauscher G, Simko I (2013). Development of genomic SSR markers for fingerprinting lettuce (*Lactuca sativa* L.) cultivars and mapping genes. BMC Plant Biol.

[11] Bhat PR, Krishnakumar V, Hendre PS, Rajendrakumar P, Varshney RK, Aggarwal RK (2005). Identification and characterization of expressed sequence tags-derived simple sequence repeats markers from robusta coffee variety ‘CxR’ (an interspecific hybrid of *Coffea canephora* x *Coffea acongensis*). Mol Ecol Notes.

[12] Jungmann L, Vigna BBZ, Paiva J, Sousa ACB, do Valle CB, Laborda PR (2009). Development of microsatellite markers for *Brachiaria humidicola* (Rendle) Schweick. Conserv Genet Resour.

[13] Vigna BBZ, Alleoni GC, Jungmann L, do Valle CB, Souza AP (2011). New microsatellite markers developed from *Urochloa humidicola* (Poaceae) and cross amplification in different *Urochloa* species. BMC Res Notes.

[14] Doyle JJ, Doyle JL (1987). A rapid DNA isolation procedure for small quantities of fresh leaf tissue. Phytochem Bull.

[15] Billotte N, Lagoda PJR, Risterucci AM, Baurens FC (1999). Microsatellite-enriched libraries: applied methodology for the development of SSR markers in tropical crops. Fruits.

[16] Thiel T. MISA — MIcroSAtellite identification tool, Version 1.0. In: MISA — MIcroSAtellite identification tool. Leibniz Institute of Plant Genetics and Crop Plant Research. 2001. http://pgrc.ipk-gatersleben.de/misa/misa.html. Accessed 21 August 2012.

[17] Untergasser A, Nijveen H, Rao X, Bisseling T, Geurts R, Leunissen JA (2007). Primer3Plus, an enhanced web interface to Primer3. Nucleic Acids Res.

[18] Creste S, Tulmann Neto A, Figueira A (2001). Detection of single sequence repeat polymorphisms in denature polyacrylamide sequencing gels by silver staining. Plant Mol Bio Rep.

[19] Esselink GD, Nybom H, Vosman B (2004). Assignment of allelic configuration in polyploids using the MAC-PR (microsatellite DNA allele counting*—*peak ratios) method. Theor Appl Genet.

[20] Clark LV, Jasieniuk M (2011). Polysat: an R package for polyploid microsatellite analysis. Mol Ecol Resour.

[21] Mateescu RG, Zhang Z, Tsai K, Phavaphutanon J, Burton Wursten NI, Lust G (2005). Analysis of allele fidelity, polymorphic information content, and density of microsatellites in a genome-wide screening for hip dysplasia in a crossbreed pedigree. J Hered.

[22] Tessier C, David J, This P, Boursiquot JM, Charrier A (1999). Optimization of the choice of molecular markers for varietal identification in *Vitis vinifera* L. Theor Appl Genet.

[23] Pritchard J, Stephens M, Donnelly P (2000). Inference of population structure using multilocus genotype data. Genetics.

[24] Falush D, Stephens M, Pritchard JK (2003). Inference of population structure using multilocus genotype data: linked loci and correlated allele frequencies. Genetics.

[25] Falush D, Stephens M, Pritchard JK (2007). Inference of population structure using multilocus genotype data: dominant markers and null alleles. Mol Ecol Notes.

[26] Evanno G, Regnaut S, Goudet J (2005). Detecting the number of clusters of individuals using the software STRUCTURE: a simulation study. Mol Ecol.

[27] Earl DA, von Holdt BM (2012). STRUCTURE HARVESTER: a website and program for visualizing STRUCTURE output and implementing the Evanno method. Conserv Genet Res.

[28] Jakobsson M, Rosenberg NA (2007). CLUMPP: a cluster matching and permutation program for dealing with label switching and multimodality in analysis of population structure. Bioinformatics.

[29] Rosenberg NA (2004). DISTRUCT: a program for the graphical display of population structure. Mol Ecol Notes.

[30] Morgante M, Hanafey M, Powell W (2002). Microsatellites are preferentially associated with nonrepetitive DNA in plant genomes. Nat Genet.

[31] Gonzalez AMT, Morton CM (2005). Molecular and morphological phylogenetic analysis of *Brachiaria* and *Urochloa* (Poaceae). Mol Phylogenet Evol.

